# Identifying Design Opportunities for Adaptive mHealth Interventions That Target General Well-Being: Interview Study With Informal Care Partners

**DOI:** 10.2196/47813

**Published:** 2023-10-24

**Authors:** Xinghui Yan, Mark W Newman, Sun Young Park, Angelle Sander, Sung Won Choi, Jennifer Miner, Zhenke Wu, Noelle Carlozzi

**Affiliations:** 1 School of Information University of Michigan Ann Arbor, MI United States; 2 Penny W Stamps School of Art and Design University of Michigan Ann Arbor, MI United States; 3 H Ben Taub Department of Physical Medicine and Rehabilitation Baylor College of Medicine Houston, TX United States; 4 Department of Pediatrics University of Michigan Ann Arbor, MI United States; 5 Department of Physical Medicine and Rehabilitation University of Michigan Ann Arbor, MI United States

**Keywords:** mHealth intervention, mobile health, behavior change, qualitative study, user adherence, behavioral messages, general well-being

## Abstract

**Background:**

Mobile health (mHealth) interventions can deliver personalized behavioral support to users in daily contexts. These interventions have been increasingly adopted to support individuals who require low-cost and low-burden support. Prior research has demonstrated the feasibility and acceptability of an mHealth intervention app (CareQOL) designed for use with informal care partners. To further optimize the intervention delivery, we need to investigate how care partners, many of whom lack the time for self-care, react and act in response to different behavioral messages.

**Objective:**

The goal of this study was to understand the factors that impact care partners’ decision-making and actions in response to different behavioral messages. Insights from this study will help optimize future tailored and personalized behavioral interventions.

**Methods:**

We conducted semistructured interviews with participants who had recently completed a 3-month randomized controlled feasibility trial of the CareQOL mHealth intervention app. Of the 36 participants from the treatment group of the randomized controlled trial, 23 (64%) participated in these interviews. To prepare for each interview, the team first selected representative behavioral messages (eg, targeting different health dimensions) and presented them to participants during the interview to probe their influence on participants’ thoughts and actions. The time of delivery, self-reported perceptions of the day, and user ratings of a message were presented to the participants during the interviews to assist with recall.

**Results:**

The interview data showed that after receiving a message, participants took various actions in response to different messages. Participants performed suggested behaviors or adjusted them either immediately or in a delayed manner (eg, sometimes up to a month later). We identified 4 factors that shape the variations in user actions in response to different behavioral messages: uncertainties about the workload required to perform suggested behaviors, concerns about one’s ability to routinize suggested behaviors, in-the-moment willingness and ability to plan for suggested behaviors, and overall capability to engage with the intervention.

**Conclusions:**

Our study showed that care partners use mHealth behavioral messages differently regarding the immediacy of actions and the adaptation to suggested behaviors. Multiple factors influence people’s perceptions and decisions regarding when and how to take actions. Future systems should consider these factors to tailor behavioral support for individuals and design system features to support the delay or adaptation of the suggested behaviors. The findings also suggest extending the assessment of user adherence by considering the variations in user actions on behavioral support (ie, performing suggested or adjusted behaviors immediately or in a delayed manner).

**International Registered Report Identifier (IRRID):**

RR2-10.2196/32842

## Introduction

### Background

With advances in mobile technology, mobile devices can deliver timely behavioral support to individuals when they go about their daily lives (eg, to think about the positive aspects of life or increase their physical activity level) [1-4]. Behavioral support is often delivered in the form of push notifications or SMS text messages to encourage users to perform healthy behaviors with minimal attention required in context [5,6]. Such efficient and cost-effective mobile health (mHealth) support has the potential to benefit informal care partners, who are faced with considerable physical and emotional stress due to their role as a caregiver [7-10]. Prior work has examined the efficacy of mHealth interventions to monitor health and deliver support to different care partners (eg, caregivers of older adults and those of patients with heart failure and dementia [7-11]). These mHealth interventions were found to increase care partners’ physical activity, control stress levels, and have a positive effect on patient outcomes [7,8,12-14].

Despite the effectiveness of mHealth interventions reported by prior research [7,8,12-14], to improve the uptake of these interventions and sustainable adherence, it is highly valuable and necessary to investigate users’ decision-making and actions in response to behavioral support. Here, decision-making and actions are referred to as the user’s thought process of perceiving and deciding whether and how to take actions in response to the behavioral support, as well as the actual actions executed. In particular, there is a need to understand their preference regarding the type of behavioral support, which they translate into actions, and how the support should be delivered to care partners. Understanding care partners’ experience with the general well-being prompts will produce insights into optimizing future tailoring and targeting of these messages for maximal care partner engagement and benefit, as well as minimal intervention burden [15-20]. This is particularly important for care partners owing to their limited attention and availability for behavioral change and heavy responsibilities in caregiving work. With more tailored and appropriate support, mHealth interventions can ensure a high level of user adherence, which reinforces trust in the interventions and avoids long-term disengagement [21]. Therefore, it is critical to deliver behavioral support at appropriate moments when the intervention is most useful and the person is most likely to be receptive [21-23].

To this end, we need to understand how *individual* messages impact care partners’ decision-making and actions and, more importantly, which factors contribute to the success or failure of the care partner to act upon different behavioral messages. Motivated by this, we adopted a user-centered approach [24] and conducted semistructured interviews with 23 care partners who had recently used the CareQOL app (an mHealth just-in-time adaptive intervention that delivers behavioral support of different health dimensions, such as physical activity and mood) for 3 months. These participants were a subset of care partners from the intervention group of a broader randomized controlled trial (RCT). The RCT study showed that the intervention group had significantly lower levels of caregiver strain, depression, and sleep-related impairment than the control group after 3 months of CareQOL app use [25]. However, little is known about how care partners perceive different messages in their daily lives while facing heavy responsibilities and, more importantly, whether and how they translated the messages into actions. Why may certain behavioral support succeed or fail to shape care partners’ decision-making and actions? Understanding these nuances in people’s actions will help us identify future opportunities for additional tailoring and optimization of behavioral support for these care partners as well as for other populations that share similar characteristics with care partners (eg, people with limited availability).

### Objective

The primary goal of this study was to investigate care partners’ decision-making and actions in response to different behavioral messages that target general well-being. Insights from this study will guide future improvements to the existing just-in-time adaptive intervention (ie, the CareQOL app), including improved adaptations and additional person-centered tailoring. Meanwhile, we aimed to provide future opportunities to improve user experience with mHealth behavioral interventions, ensuring the successful uptake and sustainable use of mHealth interventions.

In this study, we asked the following research questions:

1. How do care partners’ decision-making and actions vary in response to behavioral messages that target general well-being (eg, physical activity, sleep)?
2. What factors influence care partners’ decision-making and actions in response to behavioral support?
3. How should we incorporate people’s decision-making and different actions into the better design and evaluation of mHealth interventions?

## Methods

To understand how behavioral messages impact people’s decision-making and different actions, we conducted interviews with 23 care partners who exited from the intervention group of a larger RCT study (ClinicalTrials.gov NCT04556591) [25,12]. In this section, we first introduce the context of the broader RCT study and then describe the interview study design and procedures.

### Broader RCT Study Context and the CareQOL Intervention

The broader RCT study aimed to examine the acceptability and feasibility of the CareQOL app among 3 distinct care partner groups after 3 months of use: care partners for persons with spinal cord injury (SCI), care partners for persons with Huntington disease (HD), and care partners for persons with episodic cancer conditions that require hematopoietic cell transplantation (HCT) [25,12]. The CareQOL app was designed to promote the general well-being of the care partners. It was paired with a Fitbit (Google LLC) and asked the participants to complete 3 ecological momentary assessment questions daily, taken from the Patient-Reported Outcomes Measurement Information System [26,27]. Both Fitbit and patient-reported data were used to tailor the behavioral messages to the participants. The broader RCT preliminarily demonstrated the feasibility of the CareQOL app in care partners. The results showed that care partners in the intervention group had a significant improvement in caregiver strain and depression alleviation and sleep habits compared with the control group [25]. More information about the RCT and CareQOL app can be found in previous publications [12].

**Figure 1:**
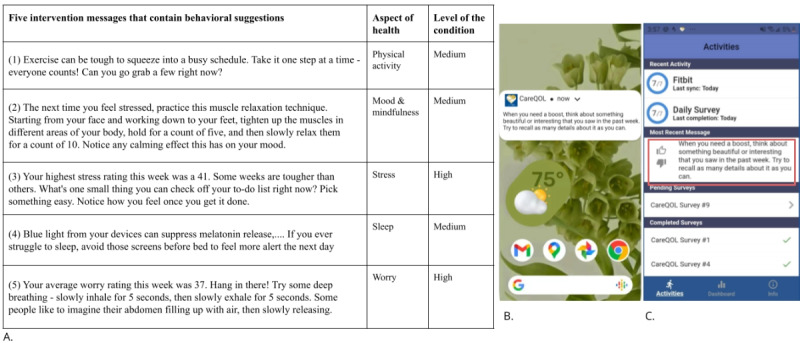
CareQOL app sends various intervention messages targeting different health dimensions, which are tailored to users’ level of the dimension from recent patient-reported outcomes. Five intervention message examples are shown in (A). CareQOL app sends periodic push notifications containing behavioral messages (B). After receiving a message, the user can revisit it in the app and rate the message (thumbs-up or thumbs-down; C).

The CareQOL app used users’ Fitbit data and ecological momentary assessment responses as tailoring variables to categorize their activity level, sleep, and thoughts or emotions (eg, sadness) into 3 levels (ie, high, medium, and low), following prior work [12]. This app used a message pool of 412 unique intervention messages that communicated about 40 specific behavioral messages in 6 dimensions of health (ie, physical activity, sleep, caregiver stress, sadness, worry, and mood and mindfulness). The design of the message pool was inspired by a previous study on tailoring an mHealth intervention for medical interns who were susceptible to mental health issues [28]. Building on the existing message pool that has been demonstrated to offer benefits to people’s mood, step counts, and sleep [28], the last author’s research team worked with different stakeholders, including care partners and clinical experts, to modify the content of messages tailored to care partners (eg, “Lots of caregivers have trouble getting enough sleep every night...”). Through an interactive process, stakeholders agreed upon the messages to help promote healthy behaviors and care partners’ general well-being. These messages offered by CareQOL include items such as increasing walking, practicing meditation or deep breathing, talking to friends, and listening to music when stressed. In Figure 1A, we offer 5 example behavioral messages containing specific suggestions; their target dimensions of health; and the level of the dimension (ie, high, medium, or low). These suggestions were delivered via push notifications on approximately half of the days during the intervention period, and the user could view the most recent one within the app (Figures 1B and 1C). In addition, participants were encouraged to rate each message by giving it either a thumbs-up (like) or a thumbs-down (dislike; Figure 1C).

### Research Design

We conducted retrospective interviews with 23 care partners who were enrolled in the intervention arm of the broader RCT [12] at the time of their completion. The goal of the interviews was to help us understand the care partners’ decision-making and actions in response to different behavioral messages.

### Interview Study Procedures

Each interview session lasted approximately 1 hour. To help the participants better recall their experiences with the CareQOL app, we showed actual user data, including the intervention messages delivered to the user with the dates of the messages received and the corresponding user’s rating (ie, like or dislike) during the interview session. For each interview, we first prepared screenshots with different messages that participants received during the RCT as “probes” to understand participants’ actions in response to the behavioral support. This type of prompting helped participants recall their experience of engaging with an intervention message and any subsequent actions in response to them [29,30]. Considering the participants’ memory, we selected 4 to 5 intervention messages for each health dimension (eg, physical activity, sleep, and mood) based on the following criteria to capture a wide range of participants’ decision-making and actions: (1) the most recent message the participant received, (2) messages that covered a different range of the participants’ physical and mental status (eg, low, medium, or high step counts), (3) messages that participants rated liked or disliked, and (4) messages that suggested different healthy behaviors (eg, deep breathing and going for a walk). In practice, we began with the 2 most recent messages and supplemented with additional recent messages that differed in the level of the condition (eg, low, medium, or high step counts), rating, or suggested behaviors. In many cases, the participants’ physical or mental status was within the same range (eg, always low step counts). For these cases, we prioritized a wider coverage of the suggested behaviors. As the interviews proceeded, we balanced the number of times that each suggested behavior was presented to participants with the recency of the message that we were asking the participant about.

In each interview, the interviewer began with questions about participants’ life experiences while participating in the intervention, their attitudes, and progress made toward developing or improving healthy behaviors. The interviewer then walked participants through each health dimension and asked about their decision-making and actions using the prepared intervention messages. The interviewer read the content of the messages aloud, the date of delivery, and the rating of the messages (if any). For each message, the interviewer encouraged the participants to recall what happened around the date of delivery, what thoughts they had about the message, and what specific actions they took. The participants were also asked about their attitudes and feedback regarding these messages. If participants did not remember the message or their actions in context, we asked them about their thoughts and willingness to follow the specific messages.

### Recruitment

We invited participants from the intervention group of the broader RCT (n=36) to participate in this interview after their 3-month use of the CareQOL app, and 23 (64%) participants completed the interview. Participants in this interview study met the requirements for the RCT and were (1) aged at least 18 years; (2) able to read and understand English; (3) currently caring for an adult (aged ≥18 years) with medically documented HD, SCI, or HCT; 4) able and willing to use their own personal devices (eg, smartphones or tablets) and the internet for this study (download and use the CareQOL and Fitbit apps); and 5) able and willing to complete all study activities including wearing a Fitbit Charge 2 throughout the study duration.

Of the 23 participants who participated in the interview, 6 (26%) were SCI caregivers, 11 (48%) were HCT caregivers, and 6 (26%) were HD caregivers; 4 (17%) participants were male, and 19 (83%) were female. The average age of the participants was 55 years (SD=11.05). Participants who opted to complete the interview provided separate informed consent for the interview. Interviews were conducted using Zoom (Zoom Video Communications Inc) with audio only, and video was turned off). Audio recordings of the interviews were saved and transcribed by the Zoom platform, with any clarifications made by the interviewer upon review of the transcripts.

### Ethical Considerations

This study was approved by the Institutional Review Board for Medical Research (IRBMED) (HUM00184455). The participants were compensated with US $25 for the completion of the interview.

### Data Analysis

The interviews were audio recorded and transcribed for data analysis. We segmented the interview data by different intervention messages and the corresponding reported actions. We generated initial codes (eg, decision-making process, action vs nonaction on the messages, actions in response to physical or mental well-being–related messages) based on our goal of investigating various actions and conducted the first round of coding. Given that participants might have a different perception about a message during the interview, as opposed to when the message was received in real time, we focused on responses where participants indicated adequate recall of the message and the associated action. In cases where participants were unable to recall a given message (or the surrounding context), we asked the participants for their general impressions with regard to the future refinement of the system. We used in vivo and descriptive coding to understand the factors influencing decision-making [29]. Meanwhile, we wrote memos on the interview data and participants’ demographic information to complement the coding analysis and help us identify factors underlying participants’ decision-making regarding their behaviors [31]. The study team met multiple times throughout the coding analysis process to iteratively refine the list of factors, reach an agreement on the identified factors, and understand each finding. The coding process was further reviewed as a form of peer debriefing to refine, develop, and organize the findings.

## Results

### Overview

The 23 participants received 1043 intervention messages containing behavioral messages over the 3-month period of the RCT, with an average of approximately 45 (SD 8.80) messages for each individual. Each participant was presented with 20 to 25 messages during the interviews. We collected 287 instances of user actions and nonactions (ie, participants received an intervention message but decided not to take any action, based on their responses in the interview), with each participant reporting 12 instances on average (SD 3.20).

Participants reported that they took actions on the message suggestions, either by following the suggested health behaviors or by adjusting the suggested behaviors. For both types of behaviors, participants took actions immediately upon receiving the message suggestion or at a later time to purposely integrate the suggestions into their daily lives. On the basis of the variations in the temporality and adaptation to the originally suggested behaviors, participants’ actions can be classified into 4 types. In Table 1, we present examples of participants’ actions through 2 specific intervention messages (ie, taking more steps or practicing meditation). Note, in some cases, that participants successfully planned and delayed their actions to a further time, such as even a month after receiving the message.

**Table 1:** Participants reported performing suggested behaviors or adjusted behaviors (“adaptation”) either immediately or in a delayed manner (“temporality”) in response to 2 behavioral messages that are about taking more steps (example 1) and practicing meditation (example 2).

Actions on the intervention message		Immediate action	Delayed action
**Message example 1: “Last week you averaged x,xxx daily steps...Can you add more steps? Reward yourself when you hit your goal.”**			
	Performing the suggested behavior	No immediate action reported by participants	“Walked with my partner at the end of the day.” [P7]
	Adjusting the suggested behavior	“Worked on the garden right away to be more active.” [P18]	“Anticipated and planned to be physically active this weekend for the scheduled volunteering work.” [P6]
**Message example 2:** **“Your daily worry rating has varied from x to x over the past month. Try meditating to relieve anxiety and help focus your mind.”**			
	Performing the suggested behavior	No immediate action reported by participants	“Encouraged by my partner and practiced meditation together a few days later.” [P5]
	Adjusting the suggested behavior	“Took five deep breaths instead right away.” [P20]	“Planned activities to hang out with friends to reduce anxiety.” [P15]

The 2 examples show that participants may take different actions in response to behavioral messages. Although no participants reported performing the suggested behaviors right away in the examples (Table 1), sometimes participants did conduct suggested activities immediately on the condition that these activities were not time-consuming and that participants had gained previous experience. For example, when one participant (P20) received a message about deep breathing, she acted on it right away:

Every time I received a suggestion asking me to take deep breaths, I would just take five deep breaths and that is it. Even if I am in my office, I would sit still to take five deep breaths.

P20

In addition, participants adopted the exact messages (eg, avoid screens at bedtime as blue light suppresses melatonin release and thereby affects sleep; Figure 1A, message 4) immediately when they could fit them into their daily lives and had resources to alter the existing behavior. One participant stopped playing on the phone after reading that message and switched to reading on a Kindle (an electronic reading device featuring a glare-free touchscreen) instead:

I used to read on my phone or my iPad at night for hours, and after receiving it [that message], I switched to my Kindle, which does not give off blue light. That [message] helps me not have that blue light exposure before bedtime.

P14

Why may participants take different actions in response to different behavioral messages? The literature has studied factors that impact nonaction and nonadherence to behavioral support (eg, higher perceived workloads and lower perceived benefits) [22]. Beyond actions versus nonaction, we identified four critical factors that also impact users’ decisions on different actions: (1) uncertainties about the workload required by the suggested behaviors, (2) concerns about one’s ability to routinize suggested behaviors, (3) willingness and ability to anticipate opportune moments for suggested behaviors, and (4) overall capability to engage with the intervention.

### Uncertainties About the Workload Required by the Suggested Behaviors

Care partners in our study lacked time for self-care and thus appeared to be more concerned about seemingly demanding tasks. For new behaviors in which participants lacked information or prior experience, the interview data showed that they either decided not to take any actions or instead performed an adjusted behavior. For example, P20 had no experience with deep breathing exercises (Figure 1A, message 5), so when she read a message suggesting deep breathing, she chose to perform a familiar relaxing behavior:

I have a chair in my bedroom that sometimes I sit there to take a break from work. That's how I used that suggestion when I saw the message, because I was not sure how to practice it [deep breathing]. It [taking some actions] made me feel that I am taking care of myself.

P20

In addition, even when participants were provided with detailed instructions about a suggested behavior, they still did not engage in these messages if they were uncertain about the actual workload taken to implement a message, for example, the effort taken to prepare mentally. For example, one participant, in response to a muscle-relaxation message (Figure 1A, message 3), told us the following:

I did not try this out, probably because it was difficult for me to stop what I was doing and get into that mental state. To me, it is more stressful rather than relaxing...I just did what I usually do, pulling into a parking lot and closing my eyes for ten minutes. That message kind of gave me a reminder, like I should get an energizer even during the day.

P5

Thus, even though this participant did not follow the message exactly, they still perceived benefits from using the intervention messages as a reminder to enhance their current healthy behaviors.

### Concerns About One’s Ability to Routinize Suggested Behaviors

Our interviews showed that our participants perceived limited benefits if they did not repeat suggested behaviors or develop a routine for them (eg, did not feel capable or were not inclined to do so). This occurred even if the behavioral messages did not ask for repetition or routinization. As a result, due to the concerns about their ability to routinize suggested behaviors, participants chose not to take any action or performed an adjusted behavior instead.

Nonaction cases were mainly seen for messages about taking more steps based on a summary of weekly or monthly performance (eg, “Last week you averaged 7,816 steps per day. Keep up the great work and you'll continue to reap health and wellness benefits!”) Such messages made the participants feel that it was necessary to develop a walking routine. Therefore, when participants felt it difficult to have a daily walking routine, they did not take any action in response to “keep up the great work.” For one participant, the perceived incapability of developing a routine was due to his ever-changing plans:

My plans got changed from day to day. It is hard for me to think of what would happen next week and I don’t think I could keep a routine to do exercise.

P6

Similarly, for P3, even though she received positive feedback on satisfying step counts, she did not intend to improve her physical activity level because “it [high step counts] was just not replicable.”

Some participants reported choosing an alternate behavior to the suggested one when they did not feel capable of integrating the suggested behavior into their lives. One participant perceived it difficult for her to pick up yoga and stick to this new activity. She tried to set up more time for running outside to relieve stress:

It was not possible to pick up a new activity [doing yoga]. I might try it once, but could not make it part of my self-care. For running, it is something I’ve already done and it helps with my stress level...I remember giving it [this message] a thumb-down but it kind of reminded me of reducing stress in my own ways.

P12

Thus, the behavioral message was successful in increasing an existing positive behavior that was similar to but not the same as the suggested one.

Overall, although the CareQOL app did not ask for building a routine for the suggested behaviors, in some cases, participants still perceived the necessity of routinizing the suggested behaviors. When participants were concerned about their capability to establish a routine, they would rather not try the suggested behavior, often resulting in nonactions on the exact messages. However, some participants showed flexibility in adapting messages to fit the existing behaviors and schedules.

### In-the-Moment Willingness and Ability to Plan for Suggested Behaviors

Immediately upon reading a message, some participants might not be willing or able to envision future moments when they could integrate the message into their daily lives. This usually resulted in the participants’ nonactions. By contrast, some participants were able to anticipate future opportune moments for suggested behaviors, and they often carried them out at a later time (delayed action), sometimes even a month after receiving the message.

Many participants reported difficulty finding the time to think about how they would implement a message at a later time, as they were too busy when they received the message. Often, this resulted in the participants’ nonactions. For example, P11 reported rarely acting on any messages because her ongoing work limited her ability to think about how to implement them:

I didn’t think they weren’t good advice, but I read something and I’m going about my day. I didn’t have the time to think, OK, “what should I do with this [message]?”

P11

It seemed that if participants did not attend to the messages immediately, they were less likely to think of taking actions at a later time. When P3 reflected on her experience with the CareQOL app in general, she realized that although she thought she might be able to go back to the messages later, these messages were usually left unattended, and, therefore, no action was taken:

[These messages were all] good ideas and if I'm feeling low, I would love to get out and walk a little bit or meditate, but I have these other things to do. So maybe I'll do it later, and then by the time later came around, it was the time to do other things.

P3

By contrast, we found that participants seemed more successful in carrying out suggested activities at a later time if they were willing and able to anticipate opportune moments upon reading the messages. In this regard, some participants reported making plans and delaying their actions to a later and more opportune moment. For example, P20 actively made plans for her daily walks upon reading physical activity–related messages (eg, “You averaged 7,209 daily steps this week!... How can you keep working that into your life?”), and she shared the experience with the research investigator:

What I usually did [after reading a message] was that I tried to look at my schedule for the week and said okay “when can I fit a walk in?” And then I just do it. I’ll make sure I’ll do it.

P20

During the interview, P16 recognized that planning for suggested activities right in context was key to her success:

[The message] said that I should take more steps during the day. I was thinking, I would try to exercise around 7 or 8 PM everyday. Things [other responsibilities] get pushed sometimes but it makes sense for me to try to schedule my exercises... And [probably since] then, when I receive a message [about physical activity], I check if I can exercise at around 8 PM and I’ll make sure I can.

P16

Interestingly, 3 participants told us that upon reading a message about improving physical activity, they reflected and envisioned future opportune moments for taking action (eg, in a month). Such opportune moments were usually when they were more available and able to establish physical activity routines (eg, after retirement). In doing so, when opportune moments occurred, the participants started executing planned actions. For example, when reflecting on a step count–related message (eg, “In the past month your average step count was 3481. How do you want next month to look? Can you improve?”). One participant expected herself to be more available and able to increase activity level after retirement and hence delayed her exercise plan until retirement:

[When I read it], I was telling myself, OK, “I will retire in a month and after that [retirement], I should get my fitness back.” On my retirement day,... I started to take a walk and now (one month after retirement), you know, I take like 7000 steps per day.

P5

Overall, we found that participants could successfully delay their actions to a later time if they were willing and able to anticipate opportune moments for suggested behaviors. On the contrary, when participants were busy with ongoing tasks or were not inclined to anticipate future opportunities right in the moment (eg, “maybe later” [P19]), they seemed less likely to attend to the messages or take any action.

### Overall Capability for Engaging With the Intervention

The aforementioned 3 factors were found to impact participants’ momentary decision-making and actions on the messages. The last factor, the overall capability to engage with the intervention, was found to impact how participants acted on nearly all behavioral messages. When disruptive events occurred to care partners (eg, the patient suddenly required intense caregiving), they prioritized caregiving activities over their own behavior change goals, and thus, their overall engagement with the app drastically decreased. Notably, even when participants returned to their normal lives and became much more capable, they were still disengaged from the intervention in general.

When facing a sudden increase in caregiving responsibilities (eg, inpatient caregiving), participants had little availability to check behavioral messages, resulting in nonaction in response to nearly all messages provided. This happened even if the suggested behaviors were not time- or effort-consuming (eg, “If a stressful situation arises, try to take your mind off it by thinking of three things in your life that are positive”). For example, at the beginning of the intervention, P17 paid close attention to behavioral messages delivered by the intervention (“I was working towards many goals recommended by this app”) and maintained a physical exercise routine (“went to the gym three times a week”). However, toward the end of the study, his wife’s condition deteriorated, and she required surgery, resulting in a sudden decrease in his overall capability for engaging with the intervention:

At the beginning of the study, I was probably giving 20% of [my attention] to her care but her condition was suddenly worse so it is much more now, maybe 80% or 90%. I did try to set aside time [for myself] but I would not be able to do so.

P17

Interestingly, once participants’ overall attention to intervention notifications decreased, it was difficult to reengage our participants. Even when some participants resumed their normal lives after a demanding disruptive event (eg, feeling more available with fewer responsibilities), they seemed already used to ignoring intervention messages and thus still did not take any action. For example, P13’s patient was rehospitalized, which required 24×7 emergent caregiving responsibility for P13. One month later, although her husband gradually recovered and her life was “finally back to normal again,” she did not pay attention to intervention notifications or try to act on the messages:

When my husband was sent home, I started to have time for myself. But I don’t think I read those suggestions as carefully as I used to be [before the patient’s rehospitalization]. I did read some messages only if I happened to be on my phone, but certainly not all of them.

P13

In a different example, due to a foot injury that lasted about 3 weeks, P6 seemed no longer attentive to intervention messages, even though he purposely tried to return to his original activity level:

I injured my foot at some point during the study. Many suggestions did not make sense to me. Because of my foot injury, I couldn’t do all that extra stuff [physical exercises].... When I felt much better, although I was trying to get my step counts back, I didn’t really count on those messages anymore.

P6

In summary, when participants experienced disruptive events that were prioritized over their behavior change goals or intervened with their self-care practice (eg, injury), participants’ overall capacity for engaging with the intervention decreased, resulting in nonaction. Interestingly, even if our participants gradually returned to their normal life situations (eg, having fewer caregiving responsibilities), they still did not resume their original level of engagement with the intervention, resulting in nonaction.

## Discussion

### Principal Findings

We found that care partners’ actions in response to different behavioral messages varied in the temporality of the action and adaptation to the suggested behaviors. The findings further identified four primary factors that influenced user adherence and decision-making for different actions: (1) uncertainties about the workload required for suggested behaviors, (2) concerns about one’s ability to routinize suggested behaviors, (3) in-the-moment willingness and ability to plan for suggested behaviors, and (4) overall capability to engage with the intervention. These 4 factors advance our knowledge of care partners’ decision-making and actions in response to behavioral support.

Our findings highlight that not only the workloads of a suggested behavior but also the uncertainties associated with it impact care partners’ decision-making. Although behavioral support should target small and incremental changes first rather than rush users to routinize a suggested behavior [32-34], our interviews show that care partners often think that setting up routines for suggested behaviors is necessary, as this can bring more benefits as opposed to performing them only once. As a result, if care partners perceive a gap between their capabilities and the necessity of developing a routine for the suggested behavior, they choose not to act on the message or improve their existing healthy behavior. Informed by our findings, mHealth interventions should strategically persuade users that trying out a new behavior at least once can yield many benefits, while encouraging them to adapt the intervention strategies (eg, altering a new behavior or improving users’ newly learned behavior) based on the user’s experience.

In the literature, self-efficacy refers to an individual’s belief in his or her capacity to execute behaviors necessary to produce specific outcomes [35]. It has long been recognized as crucial for behavioral changes, including changes in health-related behaviors [35,36]. Self-efficacy may interact with the factors identified in our study (eg, availability of in-the-moment decision-making) to influence an individual’s actions on a behavioral message. For example, individuals with higher perceived self-efficacy may be more willing to envision opportune moments in context, even if they are busy. On the contrary, a caregiver may be available to engage in behavior change, but he or she may fail to act due to low self-efficacy in making changes. In addition, our findings show that individuals’ perceived ability to work messages into daily routines impacts their decision-making process. Building on the concept of self-efficacy, our findings highlight that self-efficacy in routinizing healthy behaviors may determine individuals’ decision-making and actions. In this regard, measures of self-efficacy could be built into mHealth apps and included as outcome measures when evaluating the effectiveness of mHealth interventions [35,36].

In light of our findings, we offer and discuss 3 considerations for mHealth interventions for care partner populations and, more broadly, individuals who have less availability and capability to engage with an intervention. Our findings also inform the understanding of user adherence in the context of promoting healthy behaviors and provide messages for future research.

### Considerations for the Design and Evaluation of mHealth Interventions

#### Expanding the Understanding of User Adherence by Incorporating Variations in User Actions

Informal care partners represent a group of people who can spare less time and effort for behavioral change due to the overwhelming caregiving responsibilities. Participants in this study reported that they took different actions in response to behavioral support. In the context of mHealth interventions, user adherence is defined as “whether a person actually performs the target behavior” [20]. Aligned with this definition, a common practice for assessing user adherence is to track user performance and evaluate whether users perform target behaviors within a short time window (eg, achieving daily step count goals [37-39]). Our findings suggest that the evaluation of the target behaviors performed in a proximal time window may not fully reflect one’s adherence level. For care partners, they adjusted suggested behaviors (eg, shortened the duration or reduced the activity intensity) to try to meet similar health goals. By doing so, users perceived the benefits of the intervention and gained a feeling of adherence.

Informed by our findings, we argue that user adherence should involve a more nuanced assessment beyond binary states (ie, adherence vs nonadherence). For care partners, their status of being adherent can mean taking actions proximate to the suggested behaviors in an effort to promote healthy behaviors, even though the performed behaviors were not precisely what was suggested by the intervention. Therefore, regarding the assessment of one’s behavioral change, our findings suggest extending the notion of user adherence by incorporating various user decisions and actions and applying the extended notion to assessing behavior change. In a sense, the different types of user actions presented in our study can all be considered as adherence but perhaps at different levels. In some existing mHealth interventions, researchers defined a proximal time window for assessing users’ behavioral outcomes to be within 30 minutes [40]; otherwise, users’ actions are considered delayed. Some interventions assessed daily behavioral outcomes [37-39]. Our results show that participants’ actions in response to general well-being–related suggestions may occur by the end of the day, within several days, a week, or even months, influenced by factors such as a perceived opportune moment in the future. In our study, we did not further break down the different types of delays in user actions. Given that suggested behaviors require different resources to perform, there might be value in further investigating the appropriate proximal time windows and lenses for observing delayed behaviors based on the type of suggested behavior (eg, deep breathing vs walking). The relationship of these different levels of “adherence” with health outcomes could also be investigated, as increased health is the goal.

Although not reported by the participants in our study, there might be situations where the user takes more than one type of action (ie, an immediate action and a delayed action led up by the immediate action). Such cases may suggest a pathway of behavioral change worthy of future exploration. Building on this path, future research is needed to characterize different cases and levels of adherence and to identify more fine-grained determinants of user adherence in its extended definition. The flexibility of caregivers with regard to adapting messages as needed to fit within their schedules and goals is a strength that should be capitalized on for future mHealth development and that should also be captured in outcome assessment. The intervention system can query whether users take actions on a message and what those actions are. With such information, the system can further improve its personalized algorithm and guide users in a timely manner if the user takes a harmful action (eg, smoking). Moreover, with different levels of adherence characterized, mHealth systems can leverage this information to iterate decision rules for an intervention (ie, rules that specify which intervention option to offer based on tailoring variables [21]).

#### Identifying the Sweet Spot for User Agency Over Intervention Delivery

Caregiver partners often encounter disruptive caregiving events due to relapse or fluctuations in patients’ health status [7,12,41-43]. Previous research found a person’s external responsibility (eg, work) and other apps in addition to the examined intervention influence patients’ engagement [44-46]. Engagement is a broad concept that involves different aspects of user interaction with a system (eg, interaction with app features) [47]. As our goal was to investigate user decision-making and actions, we focused on user adherence to different behavioral messages, which represents a key part of the overall engagement. Our findings show that disruptive events negatively impact people’s overall availability and capability to engage with the intervention. A sudden decrease in one’s overall availability can have a prolonged negative impact on adherence to behavioral support. Moreover, in addition to factors that influence users’ temporal decision-making regarding individual behavioral messages, there are factors that can globally impact participants’ adherence and engagement.

In this direction, future research may investigate how users react to disruptive events or changes in contextual conditions (eg, being on a business trip or working on a paper deadline) and whether such changes result in temporary or continued impacts on nonadherence. Other factors such as individual characteristics, may also impact users’ capability and motivation to resume their original engagement levels with an intervention. Future investigations are expected to identify the factors that impact users’ resumption of engagement. What can the system do to minimize such possible negative effects? We see the potential for maintaining user adherence by increasing user agency in when to engage and when to willfully disengage. If the user feels temporarily overwhelmed by other responsibilities (eg, caregiving work) and can devote little attention to the intervention, give the user the flexibility to take a temporary pause, such as pausing the intervention delivery or reducing the frequency of behavioral support delivery. It is also critical to think of how to reengage users when their actual capability and availability resume, as by that time, they may have less motivation for behavior change. A similar concept to this is the “booster session” commonly seen in behavioral therapy, where therapists reengage patients in using an intervention program [48,49]. After disruptions due to other responsibilities, care partners may need a booster session with the mHealth intervention to reidentify the goal of behavioral change and how they might further engage in the future (eg, frequency of receiving behavioral support).

#### Facilitating in-the-Moment Decision-Making in Response to Behavioral Messages

Our findings show that the implementation of behavioral messages is more likely to occur when users are willing and able to envision future opportune moments to act. If they are unable to do so, then nonaction is more likely to occur. In this regard, if an mHealth intervention can facilitate positive decision-making (eg, planning for delayed actions rather than ignoring a message), it may be more likely that the user stays engaged and adherent to behavioral messages. However, mHealth features that can facilitate positive decision-making remain largely unexplored. Intervention notifications containing behavioral messages are often considered interruptions to the user’s ongoing activities [50-52]. Considering this, the added features for positive decision-making should be lightweight and efficient, and our findings suggest 2 possible directions for future system design.

First, mHealth interventions should communicate the workload and benefits related to behavioral support. Our findings highlight that in addition to delivering less demanding behavior support, an intervention system should also clearly communicate workload- and benefit-related information to assist user decision-making on actions. For example, the mHealth system can provide a time estimate for conducting suggested behaviors and possibly the required resources (eg, exercise equipment). In doing so, users could have better assistance in determining what type of actions they could take and how to allocate resources for taking actions or specifying delayed actions if their concurrent situations do not allow immediate action. An interesting yet challenging question arises: what helpful and necessary information is needed to support better and positive decision-making while not imposing a cognitive burden on users (eg, thinking of possible time for exercising)? In addition, how should mobile interfaces present information to users that require minimal attention in context? To avoid presenting repetitive information, the intervention app could educate users about the workload and benefits of healthy behaviors during the onboarding session. Future research may explore these directions to better support different types of actions in response to behavioral support.

Second, owing to different contextual and personal factors, it is sometimes unlikely that users will perform the suggested behaviors immediately (Table 1). In this regard, systems should assist users in identifying opportune moments and adjusted behaviors to facilitate user adherence (eg, assisting with quick planning and providing users with more activity options). Our findings further suggest that such assistance should be offered at the moment of perceiving behavioral support, as users are unlikely to attend to the intervention message later. Researchers and designers may consider adding and improving interaction features to support quick planning, identifying opportune moments with the help of calendars [53], and brainstorming possible adjusted behaviors that are “close” to suggested ones (eg, “If you do not have extra time for a walk outside, you can do chores or gardening to get more steps!”). These features may enhance users’ ability to integrate behavioral support into their daily lives. Notably, some of these features may not be new to current mHealth systems (eg, the Roadmap app encourages users to schedule positive activities [54]), but they have not been incorporated into users’ in-the-moment engagement with an intervention message [55]. Building on push notifications or SMS text messages, how should we embed such assistance through interaction designs? Before answering this question, future work is required to understand the extent of assistance that users need and can engage in context. For example, how specific could the planned activity be when users read a behavioral message in context [15,56] and how should users leverage the system to log their plans to be more accountable? The goal of providing assistance to facilitate decision-making is to offer more flexibility to users’ responses and actions. Engagement with these features (eg, quick planning) can also serve as data input to inform more adaptive intervention strategies. However, it is worth noting that future designs will need to balance the trade-off between increased system assistance and the user burden.

### Limitations and Future Work

Participants’ recall bias is a potential limitation of this study, as our participants might have missed or forgotten the details of important or distinctive events. However, we were still able to gain important insights into the participants’ decision-making and actions in response to different behavioral messages. In light of our findings, future work should collect more nuanced information about users’ actions (eg, whether they delay actions or perform adjusted behaviors) using in situ data collection methods to gain a more comprehensive understanding. We provided qualitative insights into the delays and adjustments in user actions. Future work may leverage quantitative evidence to differentiate between different levels of delay and different types of adjusted behaviors. Specifically, as the behavioral messages were presented as push notifications, it was challenging for the system to track whether users actually paid attention to a message. For the cases where participants reported not remembering a message, this could be because participants ignored that message. Although ignoring messages is beyond the scope of this study, there is potential value in future investigations of these situations, as well as a system improvement that might provide positive nudging for these behaviors.

In addition, most participants in this study were female caregivers, limiting the potential generalizability of this sample to male caregivers. However, our gender breakdown is consistent with the broader caregiver literature, in which >75% of care partners are female [57] and is representative of the overall breakdown of the broader study sample from which this pool of participants was drawn. However, it is also possible that the participants who elected to participate in this substudy may have had a more positive experience with the intervention, or they may have had fewer caregiving responsibilities and more free time in which to participate. However, the goal of this inquiry was to investigate the factors that led to the success or failure of behavioral messages and suggest future design opportunities. Even if such a bias is present in this subsample, we were able to identify novel factors that lead to the variation in user actions and suggest future directions to optimize an mHealth intervention. Moreover, in this study, we investigated informal care partners’ experience with the mHealth intervention targeting general well-being. Insights from this study might not be generalizable to other clinical populations. However, informal care partners represent people who lack the capability to engage with mHealth interventions, which means that our findings (eg, variations in user actions) yield important insights to inform the future design and evaluation of mHealth interventions.

### Conclusions

In this study, we examined how informal care partners took action in response to different behavioral messages targeting general well-being and identified the various factors underlying action or inaction to each suggested prompt. Specifically, we identified four factors that impacted user decision-making on different actions: (1) uncertainties about the workload required for suggested behaviors, (2) concerns about one’s ability to routinize suggested behaviors, (3) in-the-moment willingness and ability to plan for suggested behaviors, and (4) overall capability to engage with the intervention. Findings from this study shed light on future assessments of user adherence by considering the variations in user actions and intervention features that enhance user adherence and autonomy.

## Abbreviations

HCT: hematopoietic cell transplantation
HD: Huntington disease
mHealth: mobile health
RCT: randomized controlled trial
SCI: spinal cord injury
